# Brilliant blue G-assisted peeling of the internal limiting membrane in macular hole surgery

**DOI:** 10.4103/0301-4738.77047

**Published:** 2011

**Authors:** Prashant Naithani, Naginder Vashisht, Sumeet Khanduja, Subijay Sinha, Satpal Garg

**Affiliations:** Department of Vitreoretina and Uvea Services, Dr. R. P. Center for Ophthalmic Sciences, AIIMS, New Delhi, India

**Keywords:** Brilliant blue G, macular hole, internal limiting membrane, peeling

## Abstract

Dye-assisted internal limiting membrane (ILM) peeling and gas tamponade is the surgery of choice for idiopathic macular holes. Indocyanine green and trypan blue have been extensively used to stain the ILM. However, the retinal toxicity of indocyanine green and non-uniform staining with trypan blue has necessitated development of newer vital dyes. Brilliant blue G has recently been introduced as one such dye with adequate ILM staining and no reported retinal toxicity. We performed a 23-gauge pars plana vitrectomy with brilliant blue G-assisted ILM peeling in six patients with idiopathic macular holes, to assess the staining characteristics and short-term adverse effects of this dye. Adequate staining assisted in the complete removal of ILM and closure of macular holes in all cases. There was no evidence of intraoperative or postoperative dye-related toxicity. Brilliant blue G appears to be safe dye for ILM staining in macular hole surgery.

Internal limiting membrane (ILM) peeling, one of the most challenging procedures in vitreoretinal surgery, has been made easier with selective staining of this optically clear tissue with a number of vital dyes.[1] Indocyanine green (ICG) was one of the first dyes used for ILM peeling.[2] However, its use has recently declined due to an increasing number of reports of its toxic effect on the retina.[3,4] Trypan blue appears to be safe for intraocular use, but it stains the ILM to a lesser degree.[5] In recent times, brilliant blue G (BBG) has been introduced as an alternative to stain the ILM and appears to have no retinal toxicity in humans.[6–8] In the present study, we have investigated the efficacy and short-term safety of BBG-assisted ILM peeling in macular hole surgery.

## Materials and Methods

**Figure 1:**
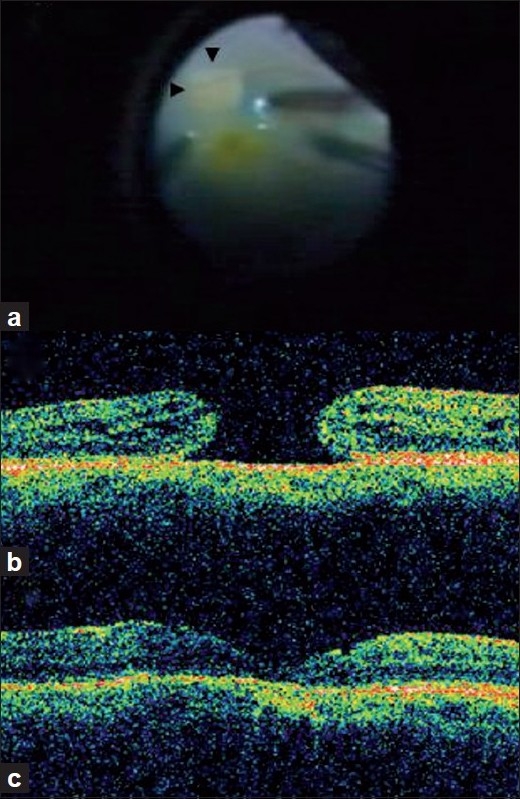
Peeling of the brilliant blue G-stained internal limiting membrane in a patient with an idiopathic macular hole (a). The excellent contrast provided between the uniformly stained internal limiting membrane (ILM) and the unstained retina exposed after ILM peeling (arrow heads) is seen. Optical coherence tomography images preoperatively, (b) and postoperatively at one week, and (c) in patient five, indicating complete macular hole closure.

After approval by the local institutional review board and ethics committee, six eyes of six patients [Table 1] with idiopathic macular holes were included in this prospective, interventional, non-comparative clinical case series. All the patients underwent a complete ophthalmic clinical examination including best corrected visual acuity (BCVA), applanation intraocular pressure, Goldmann visual fields, color and red free photographs, and optical coherence tomography (OCT) (Stratus OCT III, Carl Zeiss Meditech Inc, Jena, Germany) preoperatively, and every four weeks after surgery, for a period of 20 weeks. All the patients underwent 23-gauge three port pars plana vitrectomy (Alcon Inc., Fort Worth, Texas. USA). BBG solution (Fluoron GmbH, Neu-Ulm, Germany) was injected over the macular area and washed out after about 30 seconds. ILM staining was noted intraoperatively as good, faint or absent. The stained ILM was then peeled [Fig. 1a] with the use of an end gripping forceps. Gas fluid exchange was performed with 20% sulfur hexafluoride (SF_6_) mixture and the patients were advised prone position for one week.

**Table 1:** Baseline and follow-up clinical characteristics of all patients

Case	Age / Gender	Macular hole stage	Preoperative BCVA	BCVA at 20 weeks, postoperatively	Lens status	Postoperative macular hole status
1	60/F	3	0.15	0.40	Phakic	Closed
2	65/F	4	0.07	0.10	Phakic	Closed
3	62/M	3	0.15	0.40	Pseudophakic	Closed
4	70/F	4	0.10	0.10	Pseudophhakic	Closed
5	68/M	4	0.07	0.15	Pseudophakic	Closed
6	63/F	4	0.10	0.25	Phakic	Closed

Statistical analysis of pre- and postoperative BCVA was performed using the two-tailed Wilcoxon signed ranks test. Differences were considered significant when *P* < 0.05.

## Results

Four women and two men with a mean age of 64.6 years were included in the study. All patients were followed-up for 20 weeks postoperatively. All macular holes were completely closed in all patients in the first visit (one week) and remained closed at all subsequent four-weekly visits [Fig. 1b and c]. The mean visual acuity improved statistically and significantly from 0.11 preoperatively to 0.23 at the last follow-up postoperatively (*P* = 0.0325). Staining of the ILM was good in all cases and allowed for an easy and atraumatic removal of the ILM. The complete data on our patients is given in Table 1.

We did not observe any visual field defects in any of the patients that might have been caused by dye toxicity. There was no evidence of any retinal pigment epithelium abnormality or nerve fiber layer defect seen in the red-free photographs during any of the postoperative visits of all patients. No ocular or systemic side effects were noted.

## Discussion

Dye-related toxicity is a major concern in macular hole surgery as retinal damage caused by the dye may offset the visual gain facilitated by the successful closure of the macular hole. Reports of potential side effects of ICG point to either the dye’s photosensitizing properties[9] or to direct Müller cell damage due to alteration in the cleavage plane during ICG-assisted ILM peeling.[10] Clinical studies on trypan blue-assisted vitreoretinal surgery have revealed no toxic effect on the human retina;[5] however, t’s ability to stain ILM is inferior to ICG. A new dye, BBG has been shown as a safe tool for vitreoretinal surgery by Enaida *et al*.[6] The dye selectively stains the ILM and has no known adverse effects at present.[7,8,10] Schumann *et al*,[10] have reported that the damage caused to Müller cells during the mechanical peeling of the ILM is significantly lesser in BBG-stained specimens as opposed to ILMs stained with ICG. Although dye-related toxicity is a multifactorial process, mechanical retinal trauma is also likely to play an important role.

As the dye seeps under the detached retina surrounding the macular hole during surgery, it’s toxicity to the retinal pigment epithelial cells may affect the final visual function adversely. ICG has been seen to cause damage to the retinal pigment epithelial[3] and ganglion cells,[4] and can potentially compensate the functional gains achieved with successful macular hole surgery. In contrast, no statistically significant differences were observed in the density of retinal ganglion cells even one week after being exposed to BBG.[8]

There was absence of any visual field defect either within the area of the peeled ILM indicating trauma to the nerve fiber layer during ILM removal, or outside it indicating any dye-related toxicity in our study. None of the patients showed any retinal nerve fiber defects on red-free photographs as well. Although there were no studies documenting the postoperative duration for which BBG was retained in the eye, the absence of any visual field defects at the end of 20 weeks, pointed to the dye’s lack of toxicity to retinal elements, as investigations on ICG-related field defects reported such events within the first few follow-up visits.[8]

All the patients in our series had macular holes measuring greater than 400 μm and we attributed the successful closure rate to BBG-assisted adequate removal of the ILM. Staining of the ILM was good in all cases, providing an excellent contrast and facilitating complete removal of the membrane atraumatically. The low potential toxicity and staining nature of BBG would make it safer than trypan blue or ICG. Studies with larger patient size would be possible when the dye becomes more easily available to vitreoretinal surgeons in India, in the future.
